# Effects of Oral 5-Aminolevulinic Acid on Lipopolysaccharide-Induced Ocular Inflammation in Rats

**DOI:** 10.3390/vetsci10030207

**Published:** 2023-03-09

**Authors:** Yuya Otaka, Kazutaka Kanai, Daiki Okada, Noriaki Nagai, Yohei Yamashita, Yoichiro Ichikawa, Kazuki Tajima

**Affiliations:** 1 Department of Small Animal Internal Medicine II, School of Veterinary Medicine, Kitasato University, 35-1 Higashi 23 ban-cho, Towada 034-8628, Aomori, Japan; 2 Faculty of Pharmacy, Kindai University, 3-4-1 Kowakae, Higashiosaka 577-8502, Osaka, Japan

**Keywords:** anti-inflammatory effect, endotoxin-induced uveitis, 5-aminolevulinic acid

## Abstract

**Simple Summary:**

Uveitis causes severe vision impairment, leading to blindness. Clinically, corticosteroids are its first-line treatment to reduce inflammation, but they can cause systemic and local side effects. Recently, 5-aminolevulinic acid (5-ALA) has received attention for its reported anti-inflammatory effects. However, the effects by 5-ALA on uveitis are still insufficiently understood. Therefore, this study aims to investigate whether 5-ALA inhibits endotoxin-induced uveitis (EIU) in rats. Results showed that 5-ALA dose-dependently suppressed the clinical score of EIU, decreased aqueous humor infiltrating cells and protein concentrations, and improved histopathological scores. In particular, 100 mg/kg 5-ALA reduced the concentration of inflammatory mediators associated with the pathogenesis of EIU. Furthermore, 5-ALA suppressed expression of inducible nitric oxide synthase in an in vitro study. In conclusion, 5-ALA suppressed inflammatory mediators and showed anti-inflammatory effects on EIU. 5-ALA needs further study because it may be an alternative to anti-inflammatory agents for treating uveitis.

**Abstract:**

This study aimed to investigate the anti-inflammatory effect of 5-aminolevulinic acid (5-ALA) on endotoxin-induced uveitis (EIU) in rats. EIU was induced in male Sprague Dawley rats by the subcutaneous injection of lipopolysaccharide (LPS). During LPS injection, 5-ALA diluted with saline was administered via gastric gavage. After 24 h, clinical scores were assessed after which aqueous humor (AqH) samples were obtained. The number of infiltrating cells, protein concentration, and levels of tumor necrosis factor-α (TNF-α), interleukin-6 (IL-6), nitric oxide (NO), and prostaglandin E2 (PGE2) in AqH were measured. For histological examination, both eyes of some rats were enucleated. In vitro, a mouse macrophage cell line (RAW264.7 cells) was stimulated by LPS with or without 5-ALA. Western blot was used to analyze the expression of inducible NO synthase (iNOS) and cyclooxygenase-2. 5-ALA suppressed the EIU clinical scores, infiltrating cell number, and protein concentration while improving the histopathologic scores. In particular, 100 mg/kg 5-ALA reduced the concentrations of NO, PGE2, TNF-α, and IL-6 in AqH, similar to 1 mg/kg prednisolone. In addition, 5-ALA suppressed iNOS upregulation in LPS-stimulated RAW264.7 cells. Therefore, 5-ALA has an anti-inflammatory effect on EIU through the inhibition of the upregulation of inflammatory mediators.

## 1. Introduction

Uveitis is an intraocular inflammation of the iris, ciliary body, and choroid. Uveitis causes severe problems and even blindness, and 10–15% of all blindness in developed countries [1]. In clinical practice, corticosteroids are the first choice of treatment to control inflammation, but they may cause systemic and local side effects [2,3]. Steroid resistance or intolerance is common in many patients [4]. This is why new treatments that are safe and effective are in demand. In order to find novel remedies, a number of studies have been conducted to explore natural substances effective against uveitis [5,6], the immunology of uveitis [7], and the link between uveitis and the microbiome [8,9].

Endotoxin-induced uveitis (EIU) developed by lipopolysaccharide (LPS) injection is the animal model for acute uveitis [10]. The inflammatory response of EIU is characterized by inflammatory cell infiltration (neutrophils, macrophages, monocytes, polymorphonuclear leukocytes, and T cells) and increased protein in the aqueous humor (AqH) by the destruction of the blood–aqueous barrier (BAB). At 24 h after EIU induction, cellular infiltration and protein leakage reach their peaks [11,12]. The mechanism that causes EIU remains unknown. However, inflammatory cytokines such as tumor necrosis factor-α (TNF-α) and interleukin-6 (IL-6) are identified as playing an important role in the development of EIU [12,13,14,15]. Inflammatory mediators such as inducible Nitric oxide (NO) derived from NO synthase (iNOS) and prostaglandin E2 (PGE2) derived from cyclooxygenase-2 (COX-2) are also involved in the development of EIU [16,17].

5-aminolevulinic acid (5-ALA) is an amino acid common to plants and animals, and is also found in some foods such as fruit, vegetables, and fermented liquor [18,19,20]. 5-ALA is known to have several valuable effects. These include anti-inflammatory [21,22], antioxidant [23], antiviral [24], and anticarcinogenic effects [25]. There are a few studies investigating the anti-inflammatory effects of 5-ALA in LPS-stimulated mouse macrophage cell lines (RAW264.7 cells) and it has been reported that 5-ALA suppresses TNF-α, IL-6, NO, and PGE2 production [21,22]. Considering the potential anti-inflammatory effects, 5-ALA might be a therapeutic option for uveitis. However, the effects of 5-ALA on ocular inflammation remain unreported.

Hence, this study aimed to investigate the anti-inflammatory effect of 5-ALA and compare such effect with that of prednisolone (Pred) in EIU rats. In addition, to elucidate the anti-inflammatory effects of 5-ALA, LPS-stimulated RAW264.7 cells were used to express iNOS and COX-2.

## 2. Materials and Methods

### 2.1. Animals

A total of 140 Sprague-Dawley rats (male, 6 weeks old, 180–220 g) were used in this study. These rats were obtained from SLC (Hamamatsu, Japan). To acclimatize the animal facility at Kitasato University (Towada, Japan), these rats were kept in an air-conditioned room for 7 days. All of the experiments were carried out by the Association for Research in Vision and Ophthalmology Statement for the Care and Use of Laboratory Animals and approved by the Animal Care and Use Committee of Kitasato University (No. 20-068). Sample size calculations were performed using R, an open-source software [26], prior to the start of the study, showing that this study provided sufficient statistical power to detect a difference between groups (Table 1).

### 2.2. Reagents and Antibodies

Neopharma Japan (Tokyo, Japan) donated the 5-ALA. We purchased LPS (from Salmonella typhimurium) and Pred from Sigma-Aldrich (St. Louis, MO, USA) and COX-2 (#12282) and α-tubulin (#2125) from Cell Signaling Technology (Danvers, MA, USA), and iNOS (ab3523) from Abcam (Cambridge, UK).

### 2.3. Induction of EIU and Animal Groups

Under anesthesia with isoflurane (Mylan Inc., Canonsburg, PA, USA), 200 µg (100 µg per site) of LPS (diluted in 0.2 mL sterile saline) was administered subcutaneously to each footpad to induce EIU. In the 5-ALA group, each rat was orally administered with 1, 10, or 100 mg/kg 5-ALA diluted in 10 mL/kg saline via gastric gavage simultaneously with LPS injection. The LPS group received the same saline volume immediately before LPS injection. As a positive control, Pred (1 mg/kg) diluted in saline (10 mL/kg) was used according to the same schedule as the 5-ALA group. The control group received the same saline volume, followed by subcutaneous injection of 0.2 mL of saline without LPS. All rats were randomized into each group as shown in Table 1.

### 2.4. Clinical Uveitis Score

The rats were graded by slit lamp 24 h after EIU induction before being euthanized. Two observers, blinded to the medication group, recorded and evaluated the clinical manifestations in the left eye. Their clinical score ratings showed no difference. Uveitis score was between 0 and 4, as previously described [27,28,29]. A grade of 0 was regarded as no inflammatory reaction, 1 as discrete dilation of the iris and conjunctival vessels, 2 as moderate dilation of the iris and conjunctival vessels, 3 as intense iridal hyperemia with a flare in the anterior chamber, and 4 as the same clinical signs as grade 3 plus the presence of fibrinous exudate in the pupillary area with an intense burst in the anterior chamber (*n* = 5 in each group).

### 2.5. Quantification of Infiltrating Cells and Protein in AqH

At 24 h after LPS injection, the rats were euthanized with an overdose of isoflurane. AqH was aspirated from both eyes using a 30-gauge needle under the operating microscope and 20–30 µL/rat collected. For counting the number of infiltrating cells, AqH from the LPS and treatment groups was diluted tenfold with sterile saline, while samples from the control group were undiluted. AqH were equally suspended in Türksteine solution and cells were counted under an optical microscope using a hematocytometer (Bürker–Türk hemocytometer; Erma Inc., Tokyo, Japan). Immediately thereafter, the AqH was centrifuged (2500 rpm, 5 min, 4 °C). The supernatants were collected and then diluted 100 times with sterilized normal saline. The control group was diluted 5 times. Protein concentration in AqH was measured using a bicinchoninic acid (BCA) protein assay kit (Pierce, Rockford, IL, USA), and the absorbance was measured using an MTP-300 microplate reader (Corona Electric Co., Ltd., Ibaraki, Japan) spectrophotometrically at 540 nm. The same day of collection, the AqH was kept on ice until cell count and total protein concentration measurements (*n* = 5 in each group).

### 2.6. Histopathologic Evaluation

The intact eyes of the euthanized rats were immediately enucleated under the surgical microscope. They were fixed in 4% paraformaldehyde solution for 24 h at 4 °C and then embedded in paraffin. Subsequently, hematoxylin and eosin staining was performed on sagittal sections (3 µm) near the optic nerve head. In histopathologic evaluation, the anterior chamber and iris-ciliary body (ICB) were graded 0–3 based on a modification used previously [30]. Grade 0 was defined as normal tissue without infiltrating cells. Grade 1 was defined as the presence of dilated iris vessels and thickened iris stroma with exudate, protein, and a few scattered inflammatory cells in the anterior chamber (≤25 inflammatory cells). Grade 2 was defined as the infiltration of inflammatory cells into the iris and ciliary body stroma with a moderate number of inflammatory cells within the anterior chamber (25–50 inflammatory cells). Grade 3 was defined as heavy infiltration of inflammatory cells within the iris stroma, ciliary body, and anterior chamber (≥50 inflammatory cells); the anterior chamber also has heavy exudation with dense protein aggregation. This analysis was performed in each group on 5 rats, 10 removed eyes in total.

### 2.7. Levels of TNF-α, IL-6, PGE2, and NO in AqH

Immediately after the rats were euthanized, AqH was obtained from both eyes. A commercially available enzyme-linked immunosorbent assay kit was used to determine TNF-α, IL-6, and PGE2 in AqH (TNF-α: KE20001; Proteintech Group Inc., Rosemont, IL, USA; IL-6: #BMS625; Thermo Fisher Scientific, Waltham, MA; PGE2: 500141; Cayman Chemical Co., Ann Arbor, MI, USA). The total nitrite (NO_2_)/nitrate (NO_3)_ in AqH was measured using a NO_2_/NO_3_ colorimetric assay kit (NK05; Dojindo Molecular Technologies Inc., Kumamoto, Japan). All kits were used according to the manufacturer’s instruction (*n* = 5 in each group).

### 2.8. Cell Culture

The RAW264.7 cells were purchased from the American Type Culture Collection (Rockville, MD, USA). These cells were cultured in RPMI-1640 medium supplemented with 2 mM L-glutamine, 10% fetal bovine serum, and 1% antibiotics (penicillin (100 U/mL) and streptomycin (100 µg/mL)). The cells were then maintained at 37 °C in a controlled humidity incubator with 5% CO_2._ Cells less than 20 passages were used.

### 2.9. Cell Viability Assays

The cell viability of RAW264.7 cells was determined with the use of the Cell Counting Kit-8 (Dojindo Molecular Technologies Inc.). These cells were seeded in triplicate in 96-well plates and pre-cultured for 24 h. They were then cultured with kit reagent (10 µL/well) for 2 h, followed by treatment with 100, 500, or 1000 µM 5-ALA for 24 h. Cell viability was determined by measuring absorbance using a microplate reader.

### 2.10. Western Blot Analysis for iNOS and COX-2

Western blotting was carried out based on our previous study [31]. In brief, sample proteins were separated by 8% SDS-polyacrylamide gel electrophoresis (PAGE) The proteins were transferred onto polyvinylidene difluoride (PVDF) membranes (Clear Blot Membrane-P Plus^®^; ATTO Corporation, Tokyo, Japan). After incubation with PVDF blocker (Toyobo Corp., Osaka, Japan), they were incubated with primary antibodies to iNOS (1:200), COX-2 (1:200), and alpha-tubulin (1:1000) for 1 h at room temperature. The membranes were incubated with horseradish peroxidase-conjugated secondary antibodies (1:20,000; GE Healthcare UK, Ltd., Chalfont St Giles, UK). Incubation was followed by incubation with ECL^TM^ Plus Western Blotting Detection Reagents (Cytiva, Bio-Technology Co., Ltd., Marlborough, FL, USA). Bands were visualized using a chemiluminescence detection device (Chemi Doc^TM^ HRS+^®^; Bio-Rad Laboratories Inc., Hercules, CA, USA) and analyzed for intensity using Image Lab^TM^ software (Bio-Rad Laboratories Inc.).

### 2.11. Statistical Analysis

Data were analyzed using StatMate V (ATMS Co., Ltd., Tokyo, Japan). We used the analysis of variance and Kruskal–Wallis test to examine the parametric and nonparametric data, respectively. The Tukey test for parametric data and the Newman–Keuls test for non-parametric data were used for ad hoc comparisons between groups. All results were expressed as mean ± standard deviations (SD). Statistically significant was defined as a *p* value less than 0.05.

## 3. Results

### 3.1. Clinical Scores

**Figure 1:**
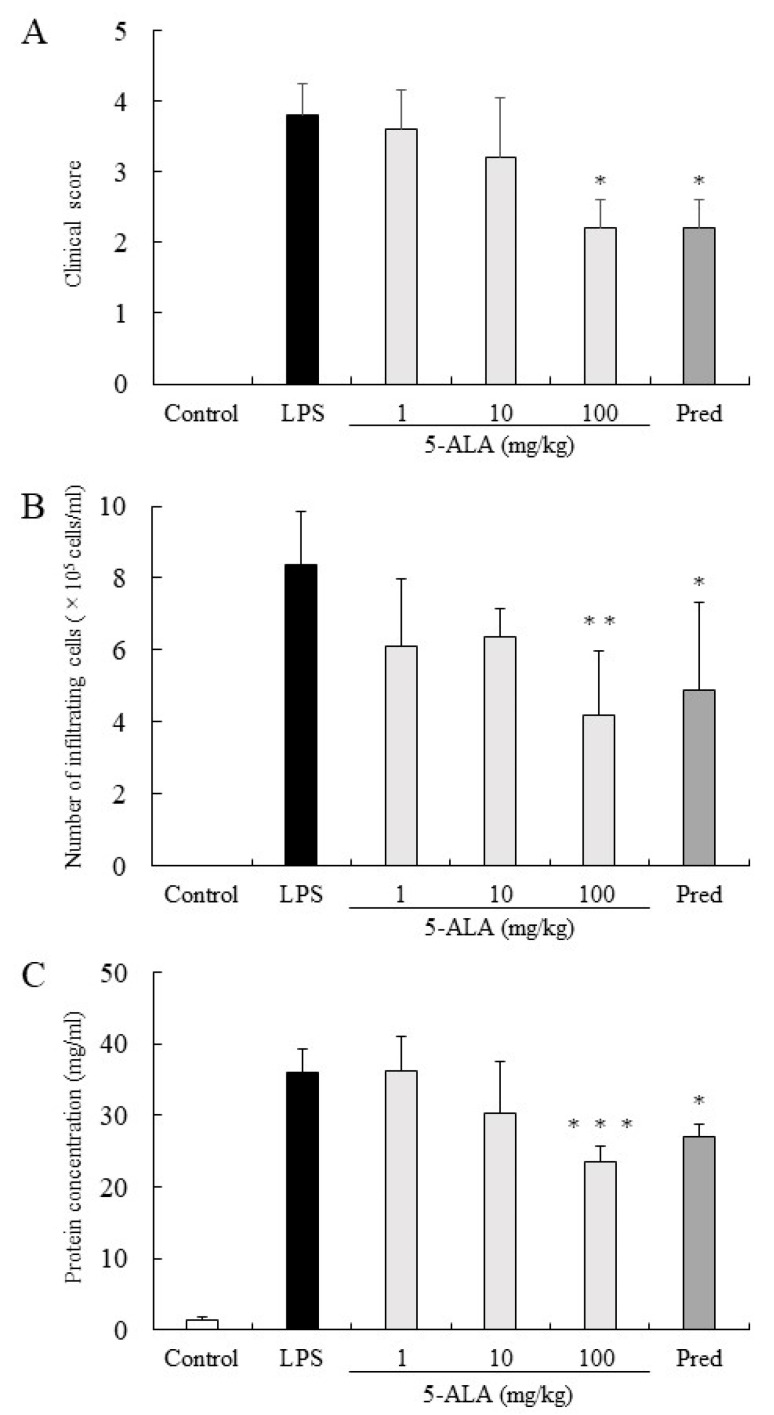
5-ALA suppressed clinical scores (**A**), the number of infiltrating cells (**B**), and protein concentration (**C**) in the AqH. Each value is expressed as the mean ± SD (*n* = 5). No clinical signs and infiltrating cells were detected in the AqH of rats without LPS (control group). * *p* < 0.05, ** *p* < 0.01, *** *p* < 0.001, compared with the LPS group. AqH, aqueous humor; LPS, lipopolysaccharide; SD, standard deviations.

The clinical score in the LPS group was 3.8 ± 0.45 (mean ± SD, *n* = 5). Rats treated with 1, 10, and 100 mg/kg of 5-ALA obtained clinical scores 3.6 ± 0.55, 3.2 ± 0.84, and 2.2 ± 0.41, respectively, decreasing dose-dependently. There were statistically significant differences between the LPS and 100 mg/kg 5-ALA groups (*p* < 0.05). Rats treated with 1 mg/kg Pred showed significantly decreased clinical scores (2.2 ± 0.41, *p* < 0.05, vs. LPS group). No difference was found between the 100 mg/kg 5-ALA and 1 mg/kg Pred groups (Figure 1A).

### 3.2. Infiltrating Cells in AqH

In the LPS group, the number of infiltrating cells in the AqH was 8.35 ± 1.52 × 10^5^ cells/mL (*n* = 5). Treatment with 1 and 10 mg/kg 5-ALA slightly reduced the number of infiltrating cells (6.1 ± 1.86 × 105 cells/mL and 6.35 ± 0.78 × 10^5^ cells/mL, respectively), with no significant difference from the LPS group. Treatment with 100 mg/kg 5-ALA and 1 mg/kg Pred significantly reduced the cell number in AqH (4.2 ± 1.76 × 10^5^ cells/mL [*p* < 0.01] and 4.9 ± 2.41 × 10^5^ cells/mL [*p* < 0.05], respectively). The effect of 100 mg/kg 5-ALA on the number of infiltrating cells in the AqH was similar to that of 1 mg/kg Pred. No infiltrating cells were found in the AqH of the control group (Figure 1B).

### 3.3. Protein Concentrations in the AqH

Protein concentration in the AqH of the LPS group was 35.90 ± 3.27 mg/mL (*n* = 5), whereas that of the control group was only 1.42 ± 0.44 mg/mL. However, treatment with 100 mg/kg 5-ALA and 1 mg/kg Pred significantly suppressed the protein concentration increase (23.50 ± 2.22 mg/mL [*p* < 0.001] and 27.10 ± 1.72 mg/mL [*p* < 0.05]), and their effects were almost the same. Moreover, treatment with 10 mg/kg 5-ALA only slightly reduced the protein concentration (30.24 ± 7.30 mg/mL), which was not significantly different from the LPS group (Figure 1C).

### 3.4. Histopathologic Evaluation

**Figure 2:**
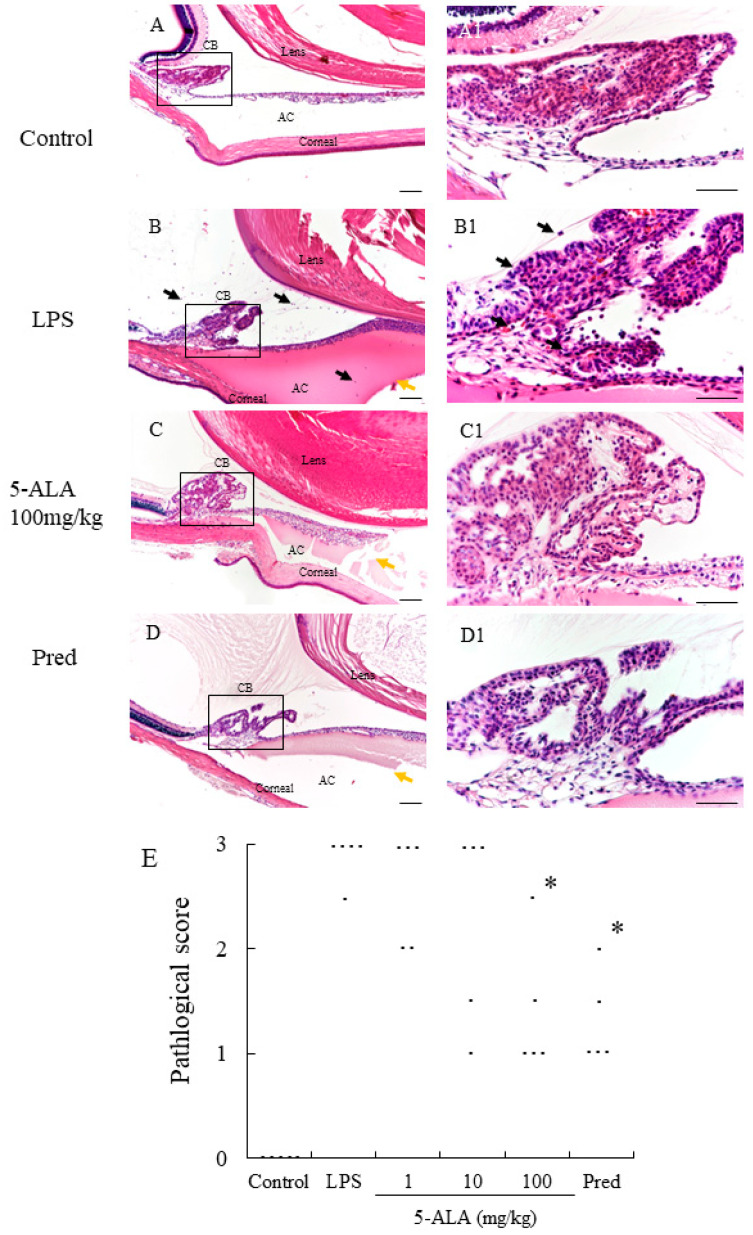
Histological evaluation treated with 5-ALA in the AC of the eye in rats. (**A**,**A1**): Control group showed normal tissue and no infiltrating cells. (**B**,**B1**): the LPS-injected rats had severe inflammatory cell infiltration within the iris stroma, CB, and AC. There was heavy exudation with dense protein aggregation in AC. Inflammatory cells and exudation with protein was reduced in the AC of rats treated with 100 mg/kg 5-ALA (**C**,**C1**) and 1 mg/kg Pred (**D**,**D1**). Black arrows mark infiltrating cells. Yellow arrows mark exudation with dense protein. Hematoxylin and eosin staining; original magnification, (**A**–**D**):×100; Bars, 100 µm, (**A1**–**D1**):×400; Bars, 50 µm; AC, anterior chamber; CB, ciliary body; (**E**) 5-ALA improved the histological grade of EIU in a concentration-dependent manner. Each dot represents the mean histopathological grading of both eyes in rats, respectively (*n* = 5). * *p* < 0.05, compared with the LPS group. EIU, endotoxin-induced uveitis; Pred, prednisolone.

In the LPS group, severe uveitis was present in the ocular tissue, and the mean histopathologic score was 2.9 + 0.22 (*n* = 5). The 100 mg/kg 5-ALA and 1 mg/kg Pred groups obtained significantly decreased histopathologic scores (1.4 + 0.65 and 1.3 ± 0.45, respectively, *p* < 0.05, vs. LPS group). The mean histopathologic scores in the 1 and 10 mg/kg 5-ALA groups were milder (2.6 ± 0.55 and 2.3 ± 0.97, respectively) than that in the LPS group, with no significant differences. The histopathologic scores of the 100 mg/kg 5-ALA and 1 mg/kg Pred groups were also not significantly different. In the control group, inflammation was not detected (Figure 2).

### 3.5. TNF-α Concentration in the AqH

**Figure 3:**
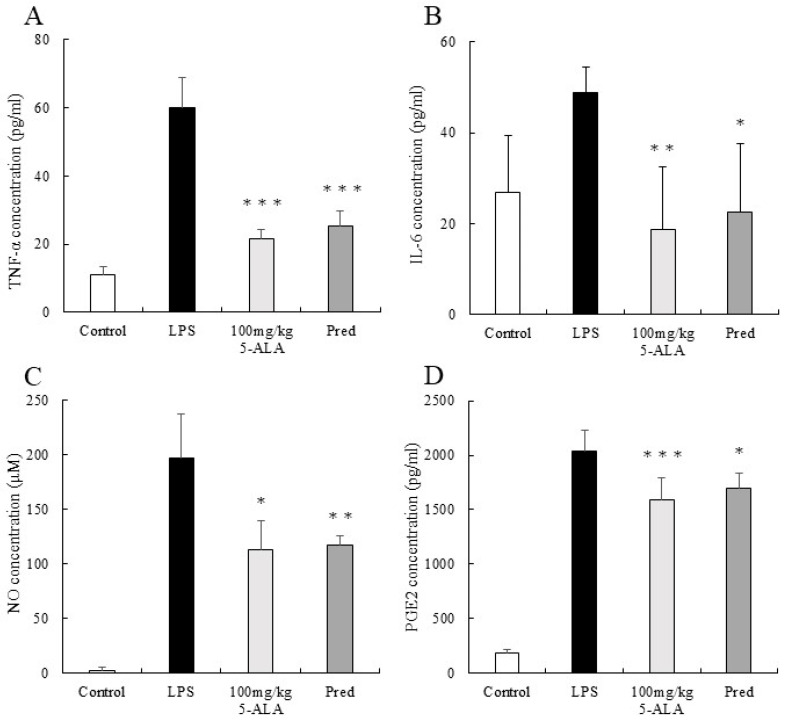
5-ALA suppressed TNF-α (**A**), IL-6 (**B**), NO (**C**), and PGE2 (**D**) concentrations. Each value represents the mean ± SD (*n* = 5). * *p* < 0.05, ** *p* < 0.01, and *** *p* < 0.001, compared with the LPS group. TNF-α, tumor necrosis factor-α; IL-6, interleukin-6; NO, nitric oxide; PGE2, prostaglandin E2.

The TNF-α concentration in AqH of the LPS group was 60.00 ± 2.46 pg/mL (*n* = 5). However, the 100 mg/kg 5-ALA and 1 mg/kg Pred groups showed significantly lower TNF-α concentrations (21.44 ± 2.76 and 25.29 ± 4.32 pg/mL, respectively, *p* < 0.001, vs. the LPS group). Furthermore, the TNF-α concentrations of the 100 mg/kg 5-ALA and 1 mg/kg Pred groups were not significantly different. The TNF-α concentration of the control group was 11.07 ± 2.46 pg/mL (Figure 3A).

### 3.6. IL-6 Concentration in the AqH

The IL-6 concentration in the AqH of the LPS group was 48.93 ± 5.46 pg/mL (*n* = 5). However, the 100 mg/kg 5-ALA and 1 mg/kg Pred groups exhibited significantly lower IL-6 concentrations (18.67 ± 13.78 pg/mL [*p* < 0.01] and 22.46 ± 15.15 pg/mL [*p* < 0.05] vs. LPS group). No significant differences were observed between 100 mg/kg 5-ALA and 1 mg/kg Pred. The IL-6 concentration of the control group was 26.84 ± 12.54 pg/mL (Figure 3B).

### 3.7. NO Concentration in the AqH

The NO concentration in the AqH of the LPS group was 197.13 ± 40.01 μM (*n* = 5), but those in the 100 mg/kg 5-ALA and 1 mg/kg Pred groups were significantly lower (112.64 ± 27.20 μM [*p* < 0.05] and 116.78 ± 8.51 μM [*p* < 0.01] vs. the LPS group). The NO concentrations in the 100 mg/kg 5-ALA and 1 mg/kg Pred groups were not significantly different. In the control group, the NO concentration was 2.13 ± 3.34 μM (Figure 3C).

### 3.8. PGE2 Concentration in the AqH

The PGE2 concentration in the AqH of the LPS group was 2041.00 ± 189.40 pg/mL (*n* = 5). The 100 mg/kg 5-ALA and 1 mg/kg Pred groups significantly decreased PGE2 concentrations (1593.83 ± 194.77 and 1699.22 ± 135.00 pg/mL, respectively) than the LPS group (*p* < 0.001 and *p* < 0.05, respectively). The PGE2 concentrations of the 100 mg/kg 5-ALA and 1 mg/kg Pred showed no significant differences. The PGE2 concentration of the control group was 189.58 ± 32.2 pg/mL (Figure 3D).

### 3.9. Cell Viability

**Figure 4:**
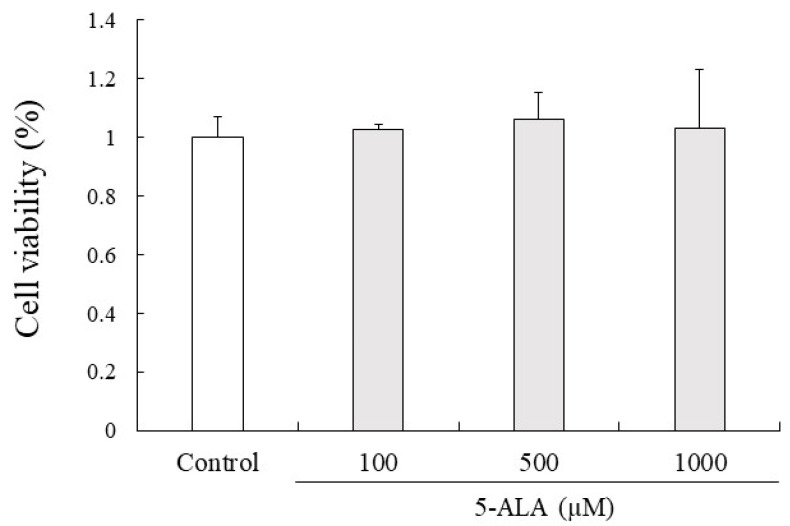
5-ALA has no effect on viability of RAW264.7 cells. Cells (0.8 × 10^4^ cells/well) were precultured for 24 h and incubated with or without 5-ALA concentrations indicated for 24 h. Data represent the mean ± SD (*n* = 3).

5-ALA had no cytotoxic effects on RAW264.7 cell proliferation at 100, 500, and 1000 µM concentrations. Given that 5-ALA showed no cytotoxicity up to 1000 µM in RAW264.7 cells, 100, 500, and 1000 µM concentrations of 5-ALA were used for in vitro studies (Figure 4).

### 3.10. iNOS and COX-2 Protein Expression in RAW264.7 Cells

**Figure 5:**
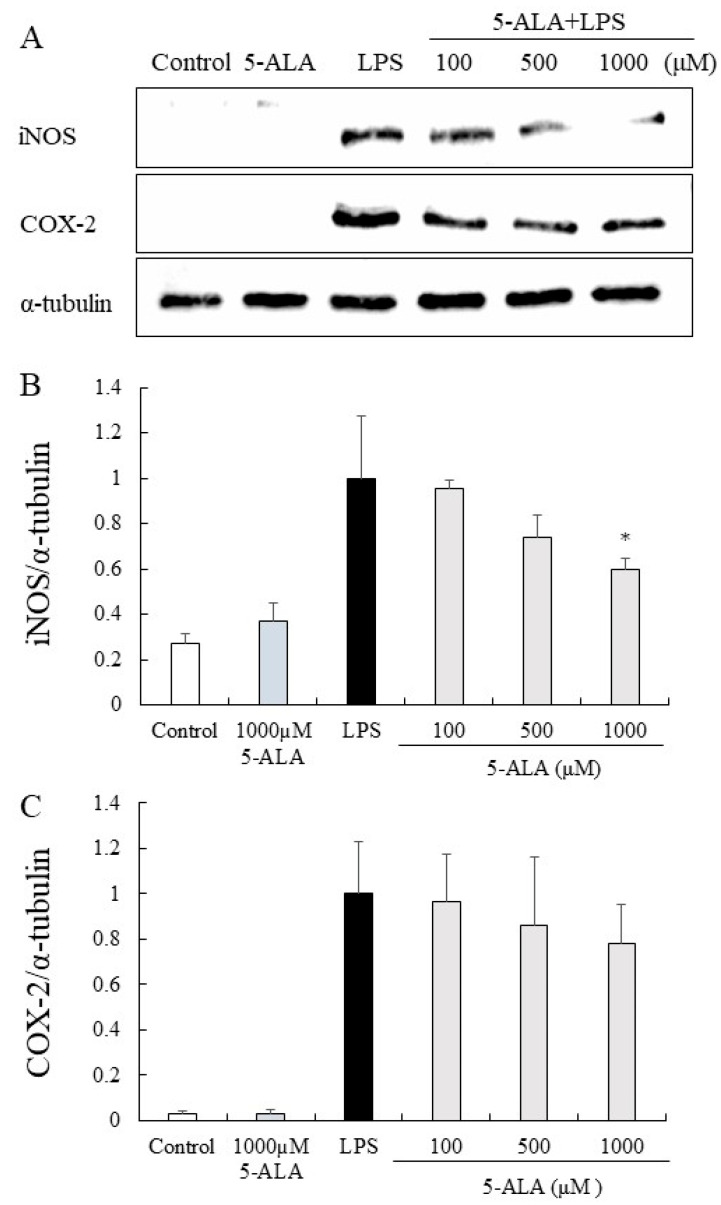
Inhibitory effects of 5-ALA on iNOS and COX-2 expression in LPS-stimulated RAW264.7 cells. With α-tubulin as a loading control, iNOS (**A**,**B**) and COX-2 (**A**,**C**) protein expression was detected by Western blot analysis. Cells were incubated with the indicated concentrations of 5-ALA and LPS for 24 h. Data represent the mean ± SD (*n* = 3). * *p* < 0.05, compared with the LPS group. iNOS, inducible NO synthase (iNOS); COX-2, cyclooxygenase-2.

The expression of iNOS and COX-2 proteins was significantly increased in the LPS group but only slightly expressed in the control group. In contrast, 5-ALA (100, 500, and 1000 µM) reduced the expression of iNOS proteins dose-dependently. Notably, 5-ALA at 1000 µM significantly reduced iNOS expression (*p* < 0.05). 5-ALA had no significant effect on COX-2 expression, however, a tendency to suppress COX-2 expression was observed (Figure 5).

## 4. Discussion

This study showed that 5-ALA significantly attenuated the intraocular inflammatory response in EIU rats, with a significant decrease in clinical scores, cellular infiltration, protein leakage into the AqH, and histopathological improvement of the characteristic manifestations of EIU. It also significantly suppressed the levels of various inflammatory markers (TNF-α, IL-6, NO, and PGE2) in the AqH. Furthermore, the anti-inflammatory effect of 1 mg/kg Pred was as strong as 100 mg/kg 5-ALA.

To elucidate the anti-inflammatory mechanisms of 5-ALA, TNF-α, IL-6, NO, and PGE2, which contribute to the pathogenesis of EIU, were measured. These pro-inflammatory mediators are released from activated inflammatory cells, especially in the ICB. [13,30,32].

TNF-α is a key inflammatory mediator, activating leukocytes, increasing neutrophil and monocyte adhesion to endothelial cells, promoting the migration of inflammatory cells into the intercellular matrix, and stimulating the synthesis of IL-6, NO, and PGE2, thereby promoting inflammatory reactions [33]. In EIU rats, TNF-α is upregulated in intraocular tissues, including the ICB. Suppression of TNF-α levels in AqH indicates reduced TNF-α expression in intraocular tissues of EIU rats [34]. TNF-α-induced uveitis in rats is associated with IL-6 [13]; therefore, TNF-α and IL-6 inhibition in the AqH suppresses uveitis development. In addition, TNF-α increases COX-2 and iNOS expression and regulates NO and PGE2 expression [16,17]. NO and PGE2 are key inflammatory mediators in the pathogenesis of EIU; when they are produced excessively in the anterior segment, vascular permeability is promoted, causing BAB breakdown in EIU [35,36,37]. In rabbits, NO and PGE2 have additive effects on EIU; inhibiting both pathways can improve uveitis [17]. In the present study, 5-ALA reduced TNF-α, IL-6, NO, and PGE2 in the AqH. Therefore, inhibiting the production of these inflammatory mediators may be the main reason for the anti-inflammatory effect of 5-ALA.

By synthesizing various cytokines and other inflammatory mediators, macrophages/monocytes may play an important role in EIU [38]. Macrophages and monocytes, followed by polymorphonuclear leukocytes and T cells, are the first inflammatory cells to enter the eye after LPS injection [39]. Thus, we used RAW264.7 cells to investigate the in vitro anti-inflammatory effects of 5-ALA. We found that 5-ALA significantly inhibited iNOS and tended to suppress COX-2 expression. Ji et al. stated 5-ALA reduced iNOS and COX-2 protein and gene expression in RAW264.7 treated with 1000 µM of 5-ALA for 1 h and then stimulated with 0.1 µg/mL of LPS for 24 h [21]. In our study, there was no significant suppression of COX-2 expression, which may be due to differences in 5-ALA treatment time. Furthermore, the anti-inflammatory effect of 5-ALA may have a more substantial effect on iNOS expression than COX-2 expression in RAW264.7 after 24 h of LPS stimulation. Further studies, including at different treatment times, would be needed to determine the effect of 5-ALA.

This study has some limitations. First, the pharmacokinetic and functional analyses of 5-ALA were not measured. Second, evaluation at different time points should have been performed; samples were only evaluated at 24 h after LPS injection. Third, given that EIU is merely an acute uveitis model, the biological effects of 5-ALA were not fully investigated, and the effects of 5-ALA in a chronic uveitis model need to be investigated. Fourth, there is a sex bias because only male rats were used in this study, and the possibility of the strength of the anti-inflammatory effect depending on sex needs to be taken into consideration [40,41].

In conclusion, 5-ALA suppresses the inflammatory process of EIU by inhibiting the production of TNF-α, IL-6, NO, and PGE2 in the anterior segment. In addition, the anti-inflammatory effect of 100 mg/kg 5-ALA on EIU is comparable to that of 1 mg/kg Pred. Broader studies are urgently needed to further investigate the potential of 5-ALA as an alternative to anti-inflammatory agents used for treating intraocular inflammatory diseases.

## Figures and Tables

**Table 1:** Number of animals used.

Group	Clinical Uveitis Score, Quantification of Infiltrating Cells and Protein in AqH	Histopathologic Evaluation	Levels of TNF-α, IL-6, PGE2, and NO in AqH
Control	*n* = 5	*n* = 5	*n* = 5
LPS	*n* = 5	*n* = 5	*n* = 5
5-ALA 1 mg/kg	*n* = 5	*n* = 5	
5-ALA 10 mg/kg	*n* = 5	*n* = 5	
5-ALA 100 mg/kg	*n* = 5	*n* = 5	*n* = 5
Pred 1 mg/kg	*n* = 5	*n* = 5	*n* = 5
Total animals (*n* = 140)	*n* = 30	*n* = 30	*n* = 80

## Data Availability

The data presented in this study are available on request from the corresponding author.
